# High prevalence rates of *Toxoplasma gondii* in cat-hunted small mammals - Evidence for parasite induced behavioural manipulation in the natural environment?

**DOI:** 10.1016/j.ijppaw.2023.01.007

**Published:** 2023-01-23

**Authors:** Miguel Pardo Gil, Daniel Hegglin, Thomas Briner, Maja Ruetten, Norbert Müller, Gastón Moré, Caroline F. Frey, Peter Deplazes, Walter Basso

**Affiliations:** a Institute of Parasitology, Department of Infectious Diseases and Pathobiology, Vetsuisse Faculty, University of Bern, Länggassstrasse 122, CH-3012, Bern, Switzerland; b Institute of Parasitology, Vetsuisse Faculty, University of Zurich, Winterthurerstrasse 266a, CH-8057, Zurich, Switzerland; c SWILD– Urban Ecology and Wildlife Research, Wuhrstrasse 12, CH-8003, Zürich, Switzerland; d Naturmuseum Solothurn, Klosterplatz 2, CH-4500, Solothurn, Switzerland; e PathoVet AG, Buckstrasse 2, CH-8317, Tagelswangen, Switzerland

**Keywords:** *Toxoplasma gondii*, *Taenia taeniaeformis*, Parasite-host manipulation, *Arvicola*, Genotyping

## Abstract

*Toxoplasma gondii* causes one of the most frequent parasitic infections in vertebrates on earth. The present study aimed to assess the occurrence of *T. gondii* infection in cat-hunted wild small mammals, and to determine the circulating *T. gondii* genotypes in cat prey. There is evidence suggesting that *T. gondii* may manipulate rodents' behaviour enhancing transmission to their definitive feline host by facilitating predation. Given that most studies focusing on rodent behavior have been performed under laboratory conditions, we tested this hypothesis in the natural environment. We analysed 157 cat-hunted wild small mammals of six different species from Switzerland. Brain and skeletal muscle samples from each animal were tested for *T. gondii* DNA by PCR, and positive samples were genotyped using a multilocus sequence typing approach, including 10 genetic markers. Additionally, to evaluate exposure to cat faeces, the presence of *Taenia taeniaeformis* metacestodes was investigated at necropsy. The prevalence of *T. gondii* in cat-hunted *Arvicola amphibius s.l.* was 11.1% (7/63), 14.6% (7/48) in *Apodemus* spp., 13.6% (3/22) in *Myodes glareolus*, 6.7% (1/15) in *Crocidura russula*, and 0% in *Microtus arvalis* (0/8) and *Sorex* sp*.* (0/1). All completely genotyped *T. gondii* parasites, exhibited the ToxoDB #3 genotype, a Type II variant. We additionally analysed 48 trap-captured *A. amphibius s.l.*, which all tested negative for *T. gondii* infection, contrasting with the higher prevalence in cat-hunted *A. amphibius s.l.* (0% vs. 11.1%; *p* = 0.0176). Furthermore, *T. taeniaeformis* was detected in both groups, indicating widespread contamination with cat faeces in the sampled areas. These results provide evidence that *T. gondii* infected rodents are at higher risk to be predated by cats and therewith support the behaviour manipulation hypothesis.

## Highlights

• High *T. gondii* infection rates in cat-hunted wild small mammals.
• ToxoDB#3 genotype circulating in wild small mammals in Switzerland.
• Challenging the behaviour manipulation hypothesis in the natural environment.

## Graphical abstract

**Figure:**
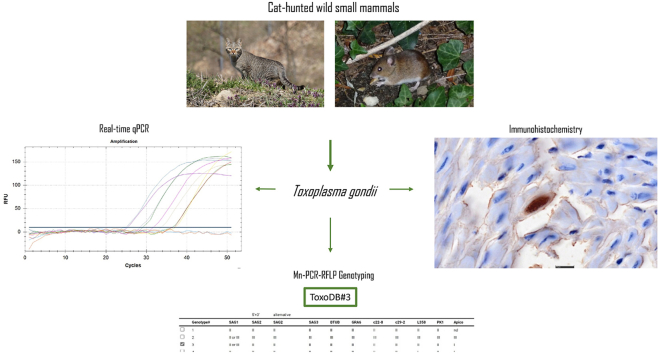

## 1 Introduction

Toxoplasmosis is one of the most frequent parasitic infections of warm-blooded animals in the world (Deplazes et al., 2016). It is estimated that up to one third of the human population is infected with this parasite (Deplazes et al., 2016). *Toxoplasma gondii* is an intracellular protozoan with an indirect life cycle including felids as definitive hosts shedding oocysts with their faeces (Deplazes et al., 2016; Dubey, 2022; Tenter et al., 2000). When the sporulated oocysts are ingested by an intermediate host, *T. gondii* undergoes two phases of asexual development. A first phase of rapidly dividing tachyzoites, associated with inflammatory and necrotic changes in different tissues, followed by a second phase of more slowly dividing bradyzoites, which are located within tissue cysts mainly in neural and muscular tissues, where they can remain for the host's lifetime (Dubey, 2022; Tenter et al., 2000). Hence, *T. gondii* has three different infectious forms, namely tachyzoites, tissue cysts, and sporulated oocysts, all of them infectious for both definitive and intermediate hosts. The hosts may become infected (i) vertically by transplacental transmission of tachyzoites, (ii) horizontally by ingestion of tissue cysts in meat and other tissues from infected intermediate hosts, or (iii) horizontally by ingestion of sporulated oocysts contaminating feed or water (Dubey, 1993, 2022; Tenter et al., 2000). The relative importance of these transmission routes varies in the different animal species. While predation is considered the main way of infection in cat populations (Afonso et al., 2007b; Tenter et al., 2000), oocysts constitute an important source of infection for humans (Boyer et al., 2011) and represent the main infection source for herbivorous intermediate hosts (Dubey, 2022).

Rodents play an important role as intermediate hosts in the life cycle of *T. gondii*, as they are major prey of felids, and thus one of their main sources of infection (Dubey, 2022; Gotteland et al., 2014). Therefore, they are considered as valuable markers for assessing environmental contamination with *T. gondii,* and infection risk for definitive hosts (Afonso et al., 2007a; Meerburg et al., 2012; Reperant et al., 2009). It is necessary to take into account potential biological, ecological and behavioural differences among rodent species, as these can significantly influence their infection level (Afonso et al., 2007a; Gotteland et al., 2014).

The domestic cat (*Felis catus*) is a fundamental species for the perpetuation of *T. gondii* (Afonso et al., 2006), as sexual reproduction of the parasite and oocysts production can only be accomplished in the intestine of felids. Domestic cat populations are growing due to increased urbanisation, and cats are considered to be invasive predators in most parts of the world (Salo et al., 2007). They are usually provided with food and shelter by their owners, in consequence, cat densities are not directly related to prey abundance, and numbers often rise well above the carrying capacity of the environment (Tschanz et al., 2011; Woods et al., 2003). In Switzerland, the cat is the most frequent companion animal roaming around, with a total number of about 1.8 million domestic cats in 2022 (https://www.vhn.ch/statistiken/heimtiere-schweiz/). In a recent study, the seroprevalence of *T. gondii* in cats with outdoor access was found to be higher (56.3%) than in those without (22.1%) (Schreiber et al., 2021). Despite the high seroprevalences, reported frequencies of oocyst shedding detection in Switzerland were much lower (below 1%), (Berger-Schoch et al., 2011; Zottler et al., 2019). It was hypothesized that there is a strong selective pressure on the parasite to develop mechanisms to increase transmission from the intermediate to the definitive feline hosts (Berdoy et al., 2000; Webster et al., 2013; Webster and McConkey, 2010). The predilection of *T. gondii* for the CNS of its intermediate hosts puts it in a privileged position to manipulate their behaviour (McConkey et al., 2013; Webster, 2007). Behavioural studies have collected abundant evidence suggesting that *T. gondii* is capable of such manipulation in the rodent intermediate hosts. Mice infected with *T. gondii* showed impaired motor performance, longer reaction times, reduced anxiety, deficits in spatial learning and memory, higher activity levels in both novel and familiar environments and most importantly, a loss of aversion to cat urine (Berdoy et al., 2000; Vyas et al., 2007; Vyas and Sapolsky, 2010; Webster, 2001, 2007). The parasite turns this innate aversion, which is present even in laboratory animals that have not been in contact with cats for generations, into a “suicidal” feline attraction (Berdoy et al., 2000; Gatkowska et al., 2012; Vyas et al., 2007). This mechanism appears to be highly specific towards cat-urine odour, as it does not affect the recognition of other predator or non-predator odours (Berdoy et al., 2000; Gatkowska et al., 2012; Vyas et al., 2007).

The aim of this study was to assess the frequency of *T. gondii* infection in cat-hunted wild small mammals in Switzerland by PCR and immunohistochemical techniques and to determine the circulating *T. gondii* genotypes in cat prey. Cats as domestic definitive hosts play a pivotal epidemiological role as amplifiers and spreaders of *T. gondii*, potentially bringing the parasite to homesteads. Furthermore, we wanted to test the hypothesis that *T. gondii* may manipulate the behaviour of rodents by comparing the prevalence of infection in cat-hunted and trap-captured European water voles (*Arvicola amphibius s.l.*).

## 2 Material and methods

### 2.1 Animals and study area

**Fig. 1:**
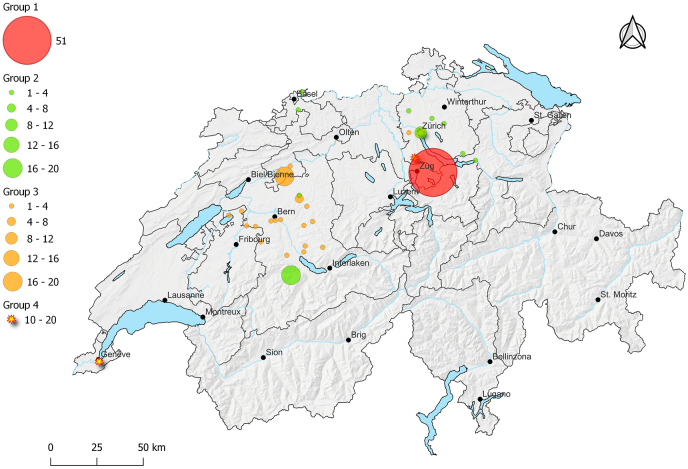
Spatial distribution of cat-hunted and trap-captured small mammals in Switzerland Map of Switzerland showing the number of sampled small mammals in each location and the distribution of the different groups used in the study. Groups 1–3: "cat-hunted"; Group 4 ″trap-captured".

We obtained small mammal samples that had been collected in Switzerland within the framework of previous projects. The sampling areas are indicated in Fig. 1 (created using the Free and Open Source QGIS, version 3.22.1 Bialowieza [http://www.qgis.org]).

The samples were divided into different groups based on their origin and mode of collection: Groups 1 to 3: cat-hunted small mammals; Group 4: trap-captured rodents.

#### 2.1.1 Group 1

These samples derived from a study aiming to investigate cat predation during springtime 2007 in the village of Finstersee (70 households, 0.25 km2, 47°10′N 8°37′E) (Fig. 1), located in the canton Zug, central Switzerland (Tschanz et al., 2011). For this purpose, cat owners were provided with plastic bags and asked to record and collect prey animals brought home by their pets (“What the cats brought home”). In many cases, only parts of the captured prey could be recovered, preventing their inclusion in the present study. We analysed 51 cat-hunted small mammals from this project, i.e., 17 European water voles (*Arvicola amphibius s.l.*, Syn. *A. terrestris, A. scherman*), 13 bank voles (*Myodes* [Syn. *Clethrionomys*] *glareolus*)*,* 16 mice (*Apodemus* spp*.*)*,* 4 greater white-toothed shrews (*Crocidura russula*) and 1 common shrew (*Sorex* sp.).

#### 2.1.2 Group 2

The samples were obtained within the framework of a SWILD (Urban Ecology & Wildlife Research, Zürich, Switzerland) project, and derived from different parts of Switzerland (Fig. 1) (Geiger et al., 2022). They were collected in plastic bags by the owners, and sent for analysis. We analysed 45 samples from the aforementioned project, collected from August 2019 to February 2021, comprising 16 *A. amphibius s.l.,* 3 *M. glareolus,* 15 *Apodemus* spp. and 11 *C. russula.*

#### 2.1.3 Group 3

This group of samples derived from the Natural History Museum of Solothurn (Solothurn, Switzerland). Small-mammals were collected as part of the project “*Zeig mir deine Maus, Katze*!” (“Show me your mouse, cat!”) (Weinberger and Briner, 2021), developed as part of the new mammal atlas of Switzerland & Liechtenstein (“Atlas der Säugetiere – Schweiz und Liechtenstein”) (Graf and Fischer, 2021). Owners were asked to collect the small mammals in plastic bags and bring them to allotted collection points, from where they were taken to the National History Museum of Solothurn. We obtained a total of 61 cat-hunted rodents, mainly from the cantons of Bern and Solothurn, Switzerland (Fig. 1) for this study. The small mammals were collected from April 2017 to March 2019 and comprised 30 *A. amphibius s.l.,* 6 *M. glareolus,* 17 *Apodemus* spp. and 8 common voles (*Microtus arvalis*)*.*

#### 2.1.4 Group 4

We obtained 48 trap-captured rodents of the species *A. amphibius s.l.* from urban and periurban areas of the city of Geneva, Switzerland, and from urban and periurban areas of the city of Zurich, Switzerland, and the nearby municipality of Rifferswil in the canton of Zurich. The rodents were trapped as part of three different projects performed in the years 2002, 2008 and 2011 (Burlet et al., 2011; Hegglin et al., 2003; Reperant et al., 2009; Stieger et al., 2002). The rodents were captured using unbaited tong traps (Hauptner Instrumente GmbH, Dietlikon, Switzerland) and Topcat traps (TOPCAT GmbH, Wintersingen, Switzerland), and they served as a comparison group to challenge the hypothesis of behaviour manipulation.

### 2.2 Parasitological examination

#### 2.2.1 Sample collection

Small mammals were conserved at −20 °C until analysis. Necropsies were performed and samples from the brain and masseter muscles were taken for *T. gondii* detection. In order to ascertain the presence of cats in all of the sampled areas, infection with *Taenia* (syn*. Hydatigera*) *taeniaeformis* (a cestode mainly transmitted by cats [Deplazes et al., 2016]), was assessed in all small mammal groups. For this purpose, dissected animals were macroscopically examined for the presence of metacestodes, particularly in the liver. The presence of *T. taeniaeformis* metacestodes was determined by opening of all lucent, round-shaped vesicles in the liver and subsequent identification of the typical strobilocerci. When a morphological identification was not conclusive, the putative metacestodes were sampled for molecular analysis.

#### 2.2.2 DNA extraction

DNA extraction from about 50 mg brain, muscle or liver/metacestode tissue, respectively, was performed using the QIAGEN DNeasy Blood & Tissue kit (QIAGEN, Basel, Switzerland) according to the manufacturer's instructions. The DNA was stored at −20 °C until use.

#### 2.2.3 Real-time qPCR

To determine the prevalence of *T. gondii* infection in cat-hunted wild mammals, a specific Taq-Man based real-time qPCR targeting the 529 bp repetitive genomic sequence of *T. gondii* (Homan et al., 2000) was used. The reaction mixture (10 μl per reaction) contained 5 μl of 2 × Mastermix (SensiFAST™ Probe NO-ROX Kit; Bioline Meridian Lifescience, Memphis, TN, USA), 0.25 μL of 20 μM-forward primer Tox-9 (5’ – AGGAGAGATATCAGGACTGTAG – 3′) and 0.25 μL of 20 μM-reverse primer Tox-11 (5’ – GCGTCGTCTCGTCTAGATCG – 3′) (Reischl et al., 2003), 0.1 μL of 10 μM detection probe Tox-HP-1 labeled with fluorescein amidite (FAM) on the 5′ end as previously described by Reischl et al. (2003) but additionally containing Black Hole Quencher 1 (BHQ1) in 3’ position as an essential element of the TaqMan hydrolysis probe (5' –(FAM)-GAGTCGGAGAGGGAGAAGATGTT-(BHQ1)-3′), 0.3 μL of 10 mM-dUTP (supplementary to dTTP included in the 2 × Mastermix) and 0.1 μL (one unit) of heat-labile uracil DNA glycosylase (UDG; both from Bioline Meridian Lifesciences). For UDG-mediated decontamination, the temperature profile included an initial 10 min incubation at 40 °C that was followed by a 5 min denaturation period at 95 °C. Subsequently, DNA amplification was achieved during 50 cycles of 10s at 95 °C and 20s at 62 °C. After each cycle, light emission by the fluorescent dye was measured at 62 °C. 2 μL of DNA template was used and the volume was completed with 2 μL of H_2_0. As negative control, 4 μL of H_2_0 were used. The amplification was performed in a CFX96 qPCR instrument (Bio-Rad Laboratories AG, Cressier, Switzerland) and analysed by applying the CFX manager, software version 1.6. Each amplification routine was conducted along with a standard curve. It was based on a 10-fold serial dilution of DNA from *T. gondii* RH strain, with tachyzoite numbers ranging from 100 to 0.01 per 1 μL (Hänggeli et al., 2022).

The standard curve, negative control as well as each tissue sample (brain and masseter muscle) were analysed in duplicate. Therefore, for each animal, four qPCR reactions were performed. One positive result out of the four qPCRs was considered as a positive animal. A limit cycle threshold value (Ct) of 40 was established.

To determine the frequency of *T. taeniaeformis* infection, a multiplex PCR to differentiate cestode species was used (Trachsel et al., 2007). *Taenia* spp. DNA positive samples were sent for Sanger-sequencing (Microsynth, Balgach, Switzerland), and the obtained sequences compared to those deposited in GenBank (https://www.ncbi.nlm.nih.gov/genbank).

#### 2.2.4 Genetic characterization of *T. gondii*

Samples showing DNA-yields with Ct values < 34 by T*. gondii* real-time qPCR were selected for further genotyping. Genetic characterization of *T. gondii* was performed by multilocus sequence typing (MLST) using nested PCR for 10 genetic markers, including SAG1, SAG2 (5′SAG2, 3′SAG2 and alt. SAG2), SAG3, BTUB, GRA6, c22-8, c29–2, L358, PK1 and Apico, as previously described (Su et al., 2006, 2010). Subsequently, PCR products were purified (DNA Clean & Concentrator-5, Zymo Research, Irvine, USA) and sequenced bi-directionally with the same primers used in the second step of the nested PCR (Microsynth, Balgach, Switzerland). The obtained marker sequences were aligned and inspected for single nucleotide polymorphisms (SNPs), digested *in silico* using the NEBcutter V2.0 programme (Vincze et al., 2003) and the RFLP profiles were analysed as described (Castro et al., 2020) in order to compare them with the *T. gondii* genotypes reported in the ToxoDB database (https://toxodb.org/toxo/app).

#### 2.2.5 Histopathology and immunohistochemistry

Masseter and heart muscle tissue from eight animals (IDs 3, 16, 21, 31, 36, 52, B42, B44) (Suppl. Material 1), which tested positive for *T. gondii* by qPCR, were fixed in formalin, embedded in paraffin, cut in 2–3 μm thin sections, mounted on glass slides and stained with hematoxylin-eosin after routine protocol. Immunohistochemical staining for *T. gondii* was performed on 2 μm thin sections, mounted on positive charged glass slides, deparaffinised and rehydrated in descending alcohol series from 100% to 70%. Endogenous peroxidase and alkaline phosphatase were blocked with Dual Endogenous Enzyme Block (Dako, Agilent Technologies, Basel, Switzerland). The slides were subsequently incubated for 1h at room temperature with a polyclonal rabbit antiserum directed against whole *T. gondii* tachyzoites at 1:2′000 dilution (Winzer et al., 2015) as primary antibody, diluted in PBS and bovine serum albumin solutions. As secondary antibody and detection system, the EnVision Peroxidase/DAB, Rabbit/Mouse was used as recommended by the manufacturer (Dako, Agilent Technologies Schweiz AG, Basel Switzerland). jhzj.

### 2.3 Statistical analysis

The observed prevalences of *T. gondii* and *T. taeniaeformis* infection in cat-hunted vs. trap-captured *A. amphibius s.l.* were compared using the Fisher's exact test (https://www.socscistatistics.com/tests/fisher)*.* Confidence intervals of obtained proportions were calculated with the Sample Size Calculators (Kohn MA, Senyak J. Sample Size Calculators [website]. UCSF CTSI. December 20, 2021. Available at https://www.sample-size.net/[Accessed November 24, 2022]).

## 3 Results

### 3.1 Frequency of *T. gondii* infection as determined by qPCR

In total, 157 cat-hunted small mammals (Groups 1–3) were tested for the presence of *T. gondii-*DNA by qPCR*,* i.e., 63 *A. amphibius s.l.,* 48 *Apodemus* spp., 22 *M. glareolus,* 15 *C. russula*, 8 *M. arvalis* and 1 *Sorex* sp. (Suppl. Material 1). We obtained qPCR positive results for *T. gondii* in 18 out of 157 animals (Table 1), yielding a prevalence of 11.5% (CI 95%: 6.9–17.5%). The prevalence of *T. gondii* in *A. amphibius s.l.* was 11.1% (7/63; CI 95%: 4.6–21.6%), 14.6% (7/48; CI 95%: 6.1–27.8%) in *Apodemus* spp., 13.6% (3/22; CI 95%: 3–35%) in *M. glareolus*, 6.7% (1/15; CI 95%: 0.2–32%) in *C. russula*, 0% in *M. arvalis* (0/8; CI 95%: 0–37%) and 0% in *Sorex* (0/1; CI 95%: 0–97.5%) (Table 2). The standard curves showed an efficiency of >78% and a regression value of >0.97. The positive DNA isolates showed Ct values ranging from 38.3 to 24.9, representing 0.01 tachyzoites/μL to 70 tachyzoites/μL, respectively (Table 1).

**Table 1:** Cat-hunted small mammals, positive for *T. gondii* DNA by qPCR.

Rodent ID	Common name	Species	Group/Location	qPCR muscle	qPCR muscle	qPCR brain	qPCR brain
2	Yellow-necked mouse	*Apodemus flavicollis*	Group 1/Finstersee, Zug	N	N	Ct: 38	N
3	European water vole	*Arvicola amphibius*	Group 1/Finstersee, Zug	Ct: 36.4	Ct:37.3	Ct: 36.5	Ct: 37.1
4	Yellow-necked mouse	*Apodemus flavicollis*	Group 1/Finstersee, Zug	N	N	Ct: 32.7	Ct: 34.1
8	Bank vole	*Myodes glareolus*	Group 1/Finstersee, Zug	N	N	Ct: 37	Ct: 36.7
9	Bank vole	*Myodes glareolus*	Group 1/Finstersee, Zug	Ct: 38.4	N	Ct: 36.7	N
10	Bank vole	*Myodes glareolus*	Group 1/Finstersee, Zug	Ct: 37.9	N	N	N
13	N/A	*Apodemus* sp.	Group 1/Finstersee, Zug	Ct: 36.9	N	Ct: 36.5	N
16	N/A	*Apodemus* sp.	Group 1/Finstersee, Zug	Ct: 36.6	N	N	N
21	European water vole	*Arvicola amphibius*	Group 1/Finstersee, Zug	Ct: 30	Ct: 30	N/A	N/A
28	N/A	*Apodemus* sp.	Group 1/Finstersee, Zug	Ct: 38	Ct: 37	N/A	N/A
31	European water vole	*Arvicola amphibius*	Group 1/Finstersee, Zug	N	Ct: 35.4	N	N
32	Greater white-toothed shrew	*Crocidura russula*	Group 1/Finstersee, Zug	Ct: 36.6	Ct: 37.4	N	N
36	European water vole	*Arvicola amphibius*	Group 1/Finstersee, Zug	N	N	Ct: 29.5	Ct: 30.2
52	European water vole	*Arvicola amphibius*	Group 1/Finstersee, Zug	N	N	Ct: 38.3	N
55	Bank vole	*Myodes glareolus*	Group 1/Finstersee, Zug	Ct: 38.6	Ct: 39.5	Ct: 26.6	Ct: 27.6
71	Yellow-necked mouse	*Apodemus flavicollis*	Group 1/Finstersee, Zug	N	Ct: 37.7	N	N
B42	European water vole	*Arvicola amphibius*	Group 3/Oey, Bern	Ct: 27.5	Ct: 27.4	Ct: 28.5	Ct: 28.5
B44	European water vole	*Arvicola amphibius*	Group 3/Oey, Bern	Ct: 24.8	Ct: 24.4	Ct: 33.3	Ct: 33

N: Negative; N/A: not applicable; Ct: cycle threshold.

**Table 2:** Prevalence of *T. gondii* infection in cat-hunted small mammals, determined by qPCR on skeletal muscle and brain tissues.

Common name	Species	Prevalence (%)
European water vole	*Arvicola amphibius*	11.1 (7/63)
N/A	*Apodemus* spp.	14.6 (7/48)
Bank vole	*Myodes glareolus*	13.6 (3/22)
Greater white-toothed shrew	*Crocidura russula*	6.7 (1/15)
Common vole	*Microtus arvalis*	0 (0/8)
Common shrew	*Sorex* sp*.*	0 (0/1)

N/A: not applicable.

None of the 48 trap-captured *A. amphibius s.l.* was positive by qPCR, resulting in a prevalence of 0% (0/48; CI 95%: 0–7.4%). Therewith the prevalence in the group of the cat-hunted *A. amphibius s.l.* was significantly higher (*p* = 0.0176, Fishers's exact test).

### 3.2 Genetic characterization of *T. gondii* DNA positive samples

Three *T. gondii* DNA isolates, obtained from two cat-hunted *A. amphibius s.l.* (IDs B42, B44) and one cat-hunted *M. glareolus* (ID 55) could be successfully genotyped at all 10 allele markers. In two further DNA isolates, derived from one *A. amphibius s.l.* (ID 36) and one *Apodemus flavicollis* (ID 4), sequences from only 9/10 and 7/10 alleles, respectively, could be obtained (Table 3). Only those DNA isolates with ≥1.8 tachyzoites/μL could be completely or almost completely genotyped. The three entirely genotyped isolates displayed a ToxoDB#3 genotype, corresponding to the clonal Type II lineage (Table 3). Alleles observed in DNA isolates from *A. amphibius s.l.* (ID 36) and *A. flavicollis* (ID 4) displayed type II sequences except for the Apico marker (in both isolates) and the GRA6 marker (in *A. flavicollis* isolate), which displayed a type I sequence. We observed a recurring SNP in the SAG3 marker in three samples (IDs 55, B42, and B44). The SNP occurred in the position 187 and it implied a substitution of thymine for adenine. We observed another SNP in the C29-2 marker in the sample ID 36, which occurred in the position 24 and implied a substitution of guanine for thymine. All other markers from all samples were 100% identical to GenBank sequences of *T. gondii* ME49.

**Table 3:** PCR-RFLP genotyping of *T. gondii* isolates from cat-hunted wild small mammals.

Rodent ID	Host species	Group/Location	Tissue	PCR-RFLP allele markers												RFLP-ToxoDB genotype
				SAG1	3′-SAG2	5′-SAG2	Alt.SAG2	SAG3	BTUB	GRA6	C22-8	C29-2	L358	PK1	Apico	
36	*Arvicola amphibius*	Group 1/Finstersee, Zug	Brain	II/III	II	I/II	II	II	II	II	II	II	II	x	I	x
55	*Myodes glareolus*	Group 1/Finstersee, Zug	Brain	II/III	II	I/II	II	II	II	II	II	II	II	II	I	**#**3
4	*Apodemus flavicollis*	Group 1/Finstersee, Zug	Brain	II/III	II	I/II	II	x	II	I	x	x	II	II	I	x
B42	*Arvicola amphibius*	Group 3/Oey, Bern	Brain	II/III	II	I/II	II	II	II	II	II	II	II	II	I	**#**3
B44	*Arvicola amphibius*	Group 3/Oey, Bern	Masseter muscle	II/III	II	I/II	II	II	II	II	II	II	II	II	I	**#**3

Note: GenBank accession numbers of the marker sequences obtained in this study: 3′SAG2:OQ102531-102535; Apico: OQ102536-102540; BTUB: OQ102541-102545; c22-8:OQ102546-102549; c29-2: OQ102550-102553; L358: OQ102554-102558

x: not amplified

### 3.3 Frequency of *Taenia taeniaeformis* infection

In 120/157 cat-hunted small mammals (Suppl. material 1) the liver was available for examination. Liver lesions/metacestodes were observed in 30 small mammals. Strobilocerci of *T. taeniaeformis* were macroscopically identified in 26 animals from two of the six small mammal species (i.e., 25 *A. amphibius s.l.*, 1 *Apodemus* spp.), belonging to all four sample groups (Table 4, Suppl. Material 2). In four cases, in which a macroscopical examination was not conclusive, a multiplex PCR for cestodes and subsequent genotyping was performed. In two of these samples (i.e., 1 *A. amphibius s.l.*, 1 *M. arvalis*) amplicons with 99.15% and 99.6% identity with *T. taeniaeformis* (JQ663994) GenBank sequences were obtained. The observed prevalences of *T. taeniaeformis* in the cat-hunted small mammals were 22.6% (12/53; CI 95%: 12.3–36.2%) in *A. amphibius s.l.*, 12.5% (1/8; CI 95%: 0.3–52.7%) in *M. arvalis,* 2.4% (1/42; CI 95%: 0.1–12.6%) in *Apodemus* spp., 0% in *M. glareolus* (0/15; CI 95%: 0–21.8%), 0% in *C. russula* (0/1; CI 95%: 0–97.5%), and 0% in *Sorex* sp. (0/1; CI 95%: 0–97.5%) (Table 4). The observed prevalence of *T. taeniaeformis* in the trap-captured group of rodents (*A. amphibius s.l.*) was 29.2% (14/48; CI 95%: 17–44%) (Table 4). The prevalence for *T. taeniaeformis* in *A. amphibius s.l.* was slightly higher in the trap-captured group (29.2%) than in the cat-preyed groups (22.6%; *p* = 0.5, Fisher's exact test).

**Table 4:** Prevalence of *Taenia taeniaeformis* in cat-hunted and trap-captured wild small mammals.

Small mammal group	Species	Prevalence of *T. taeniaeformis* (%)	No positive/n tested
Groups 1,2,3 (cat-hunted)	*Arvicola amphibius*	22.6	12/53
	*Apodemus* sp.	2.4	1/42
	*Myodes glareolus*	0	0/15
	*Crocidura russula*	0	0/1
	*Microtus arvalis*	12.5	1/8
	*Sorex*	0	0/1
Group 4 (trap-captured)	*Arvicola amphibius*	29.2	14/48

### 3.4 Histopathology and immunohistochemistry

**Fig. 2:**
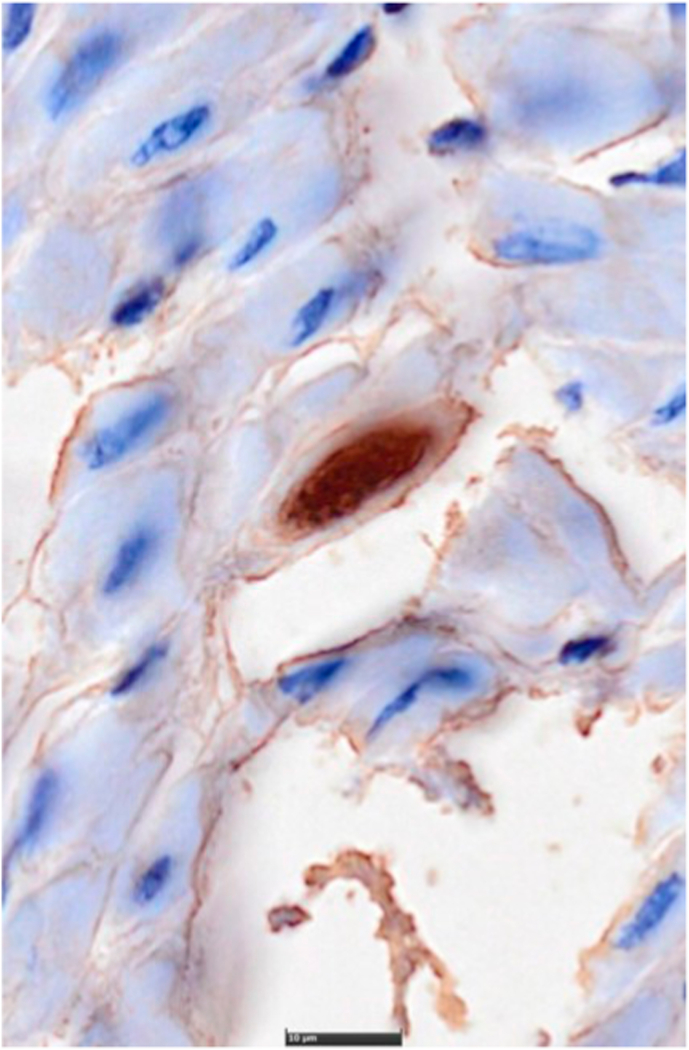
Immunohistochemical stained histological section of the heart of a European water vole (*Arvicola amphibius s.l.*) (ID B42) showing a *T. gondii* tissue cyst measuring ∼20 μm.

Eight tissue samples (i.e., masseter and heart muscle) from small mammals positive for *T. gondii* by qPCR were analysed by immunohistochemical staining for *T. gondii*. Parasite stages could be observed in tissue samples from two *A. amphibius s.l.* (IDs B42 and B44) (Fig. 2). *Toxoplasma gondii* stages were found in heart samples from both animals and in masseter sample from one animal (ID B44). They presented as intact cysts within the myofibers measuring ∼20 μm in diameter, or as free tachyzoites surrounded by numerous neutrophils and lymphocytes. Those animals showed low Ct values both for brain and masseter tissues (Table 1). Not all positive rodent samples could be analysed in histopathology due to insufficient organ tissue or bad conservation state of the available tissue, as a result of long conservation periods at −20 °C.

## 4 Discussion

In this study, we assessed the frequency of *T. gondii* infection in cat-hunted wild small mammals by molecular methods and determined the circulating *T. gondii* genotypes in cat prey. Several studies have described the level of *T. gondii* infection in wild animals (recently reviewed by Dubey, 2022); however, this is one of the first studies where the prevalence of *T. gondii* infection has been studied in cat-hunted wild small mammals. This is important, as cats play a pivotal epidemiological role as amplifiers and spreaders of *T. gondii* by hunting, bridging the sylvatic and domestic cycles of the parasite and therefore bringing the parasite closer to people's homes. In addition, we wanted to test the manipulation hypothesis, according to which *T. gondii* manipulates the behaviour of its rodent host enhancing transmission to its definitive feline host, thereby ensuring completion of its life cycle. For this purpose, we tested a group of trap-captured *A. amphibius s.l.*, which served as a comparison group to challenge the manipulation hypothesis.

### 4.1 Frequency of *T. gondii* infection in cat-hunted small mammals and circulating genotypes

Cats are fundamentally hunters of small animals (Geiger et al., 2022; Tschanz et al., 2011). We obtained a total of 157 cat-hunted small mammals from six different species with different ecological requirements. This indicates that domestic cats not only hunt in gardens, yards or grasslands, where water voles *A. amphibius s.l.* (n = 63), common voles *M. arvalis* (n = 8), and different types of shrews like *Sorex* sp. (n = 1) or *C. russula* (n = 15) may be found, but also in forests, as is showcased by the presence of bank voles *M. glareolus* (n = 22) among the brought-home-prey.

Previous studies have shown that the behavioural (e.g., fossorial vs. non-fossorial species) and ecological (e.g., forest vs. grassland vs. domestic habitat) differences among rodent species significantly influence their *T. gondii* infection level (Afonso et al., 2007a, 2007b; Gotteland et al., 2014). According to these studies, grassland fossorial species such as *A. amphibius s.l.* are considered to be at higher risk of *T. gondii* infection than non-fossorial species such as *Apodemus* spp. or *M. glareolus,* as they live in burrows in closer contact with potentially contaminated soil (Afonso et al., 2007a). This contrasts with our findings of similar prevalences in surface species of *Apodemus* (14.6%) and *M. glareolus* (13.6%) and in *A. amphibius s.l.* (11.1%). However, in contrast to most previous studies, the small mammals included in our study were hunted by cats, and therefore a bias was established from the onset. There is a high individual variability in hunting activity in cats (Fitzgerald and Turner, 2000; Tschanz et al., 2011; Woods et al., 2003), but the factors that determine the hunting strategy of domestic cats are unknown (Tschanz et al., 2011). A preference for the water vole *A. amphibius s.l.* has been observed, as it constitutes the main prey of the cats where it is abundant (Fitzgerald and Turner, 2000). Some of the projects that provided the small mammals for this study confirmed this preference (Tschanz et al., 2011; Weinberger and Briner, 2021).

We observed variability in the proportion of infected small mammals among different species, as has been reported in different studies worldwide (reviewed by Dubey, 2022) (see summary in Table 5). We compared infection rates with those studies that focused on the detection of *T. gondii* DNA in animal tissues, as serological investigations may yield higher prevalences (Glor et al., 2013). Higher infection rates than the ones reported worldwide were obtained, with a few exceptions (Table 5). Our prevalence of 11.1% in *A. amphibius s.l.* was higher than in any previous reports. High prevalences have been reported for *Apodemus sylvaticus* in two studies in England, with 34.9% (Bajnok et al., 2015) and 40.7% (Thomasson et al., 2011). All other studies worldwide reported lower prevalences in *Apodemus* spp. compared to our study (14.6%). Also, the observed prevalence for *M. glareolus* (13.6%) in this study was higher than previously reported, which is also the case for *C. russula* (6.7%). Regarding *M. arvalis*, higher prevalences have been reported, but the limited number of samples available in our study (n = 8) makes it difficult to draw conclusions. This is also applicable to the sole available sample of *Sorex* sp., which was negative.

**Table 5:** Prevalence of *T. gondii* in wild-caught rodents worldwide, determined by molecular methods, reported from 2009 to 2022.

Host	Location	No. positive/no. tested	% positive	PCR (target gene)	Reference
Striped field mouse (*Apodemus agrarius)*	Istarske Toplice, Croatia	1/43	2.3	RT-PCR (529bp)	Ivovic et al. (2019)
Striped field mouse (*Apodemus agrarius)*	Korea (South)	1/578	0.1	PCR-RFLP (GRA 5)	Hong et al. (2014)
Wood mouse (*Apodemus sylvaticus)*	Czech Republic	1/22	4.5	PCR (TGR1E)	Machačová et al. (2016)
Wood mouse (*Apodemus sylvaticus)*	Podgorje, Slovenia	1/31	3	RT-PCR (529bp)	Ivovic et al. (2019)
Wood mouse (*Apodemus sylvaticus)*	Spain	0/265	0	N-PCR (ITS1)	Fernández- Escobar et al. (2020)
Wood mouse (*Apodemus sylvaticus)*	England, United Kingdom	84/206	40.7	PCR (SAG1)	Thomasson et al. (2011)
Wood mouse (*Apodemus sylvaticus)*	England, United Kingdom	44/126	34.9	N-PCR (SAG1, SAG2, SAG3, GRA6)	Bajnok et al. (2015)
Yellow-necked mouse (*Apodemus flavicollis)*	Austria and Germany	6/222	2.7	PCR (18S rRNA)	Waindok et al. (2019)
Yellow-necked mouse (*Apodemus flavicollis)*	Czech Republic	2/265	0.75	PCR (TGR1E)	Machačová et al. (2016)
Yellow-necked mouse (*Apodemus flavicollis)*	Podgorje, Slovenia	1/16	6.2	RT-PCR (529bp)	Ivovic et al. (2019)
Bank vole (*Myodes glareolus*)	Tanap, Slovakia	1/1	100	PCR (TGR1E)	Turčeková et al. (2014)
Bank vole (*Myodes glareolus*)	Spain	0/29	0	N-PCR (ITS1)	Fernández- Escobar et al. (2020)
Common vole (*Microtus arvalis)*	Western Austria	2/264	0.7	PCR B1	Fuehrer et al. (2010)
Common vole (*Microtus arvalis)*	Lubelskie, Poland	6/70	8.6	PCR B1	Sroka et al. (2019)
Common vole (*Microtus arvalis)*	The Netherlands	1/24	4.2	RT-PCR (529bp)	Meerburg et al. (2012)
Water vole (*Arvicola amphibius*)	Western Austria	4/86	4.6	PCR (B1)	Fuehrer et al. (2010)
Greater White toothed shrew (*Crocidura russula*)	The Netherlands	2/102	2	RT-PCR (529bp)	Meerburg et al. (2012)

The higher infection rates observed herein might be explained by the fact that our samples were obtained from cat-hunted small mammals. Experimental studies have reported behavioural changes in small mammals infected with *T. gondii*, which may make them more prone to predation (Berdoy et al., 2000; Vyas et al., 2007; Vyas and Sapolsky, 2010). This implies that *T. gondii* infected small mammals would be easier prey to cats than their non-infected counterparts. Most of our positive samples derived from the Group 1 of cat-hunted small mammals (Table 1); it could be argued that this might be caused by a larger cat population in the study site (Finstersee). However, this was not the case, as the village of Finstersee has an incidence of cat ownership that corresponds to the national average of approximately one cat for every three households (Tschanz et al., 2011). It could also be argued that the other cat-hunted small mammal groups (Group 2 and 3) came from areas with a lower presence of cats. While the cat density is hard to analyse in such a heterogeneous group of samples, the presence of *T. taeniaeformis* in all animal groups confirms the exposure to cats in all of those areas. Ideally, our control group of trapped *A. amphibius s.l.* would have come from the same sites as the cat-hunted mammals. However, as we made use of mammals collected for other studies, this was not possible. Future studies will have to address this limitation and include cat-hunted and trap-captured animals collected over the same period at the same sites.

All three fully and two of the three partially genotyped *T. gondii* isolates displayed a ToxoDB#3 genotype, which belongs to the clonal Type II lineage. This is one of the most frequently found genotypes circulating amongst small mammals and other animals in Europe and Switzerland (Berger-Schoch et al., 2011; Fernández-Escobar et al., 2022; Galeh et al., 2022; Shwab et al., 2014; Spycher et al., 2011). It is to mention, that a recurring SNP in the position 187 of the SAG3 marker was observed. The same SNP has also been observed in other ToxoDB#3 circulating strains of other animal species in Switzerland (Scherrer et al. in preparation), suggesting that this is a frequent genotype variant circulating in Switzerland. The incompletely genotyped *A. flavicollis* isolate differed from the ToxoDB#3 genotype in that it had a type I sequence for the GRA6 marker.

### 4.2 Frequency of *Taenia taeniaeformis* infection

The cestode *T. taeniaeformis* develops to its adult and patent stage almost exclusively in the cat (Deplazes et al., 2016; Reperant et al., 2009), which renders cats the main source of transmission. Rodents act as intermediate hosts, in which *T. taeniaeformis* causes life-long infections (Burlet et al., 2011), making the parasite more prevalent in older animals. *Taenia taeniaeformis* was found in three (i.e., *A. amphibius s.l.*, *Apodemus* spp., *M. arvalis*) out of the six small mammal species subject to study. *Arvicola amphibius s.l.* has been found to be a frequent intermediate host of the parasite (Burlet et al., 2011). High prevalences have been previously described in Switzerland, with a 12.1% prevalence in the city of Zurich (Stieger et al., 2002), a 23.4% prevalence in the canton of Zurich (Burlet et al., 2011) and a 44.3% prevalence in adult *A. amphibius s.l.* in the canton of Geneva (Reperant et al., 2009). In accordance with these findings, we observed a higher presence of *T. taeniaeformis* in *A. amphibius s.l.* than in the other affected rodent species (Table 4, Supplementary material 2). This might be due to the longer lifespan of *A. amphibius s.l.* compared to the other species, and due to its specific ecological requirements. It is a fossorial species that lives in burrows and is therefore always in contact with soil (Gotteland et al., 2014); the loose soil of the burrows of *A. amphibius s.l.* constitutes an ideal place for the cats to defecate, as they like to bury their faeces in loose ground (Burlet et al., 2011).

The fact that *T. taeniaeformis* was found in all three different groups of cat-hunted small-mammals and also in the trap-captured group of rodents, highlights the presence of domestic cats and their faecal contamination in all of the sampling areas included in the study. It is worth mentioning the high prevalence (29.2%) in the *A. amphibius s.l.* trap-captured group, which indicates a considerable presence of cats in the sampled area.

### 4.3 Manipulation hypothesis

It is difficult to evaluate the manipulation hypothesis in the natural environment since *T. gondii* infection in small mammals involves the complex interplay between biological, spatial, and ecological factors (Dabritz et al., 2008; Gotteland et al., 2014). This includes factors such as climate and oocyst survival, different susceptibility of small mammal species to infection, diverse patterns of transmission and clinical outcomes.

As previously mentioned, there is abundant evidence suggesting that *T. gondii* is capable of manipulating the behaviour of its rodent intermediate host enhancing transmission to its feline definitive host. Given that most studies have been performed with rodents (mainly *Mus musculus* and *Rattus* spp.) maintained under laboratory conditions, the objective was to test this hypothesis in the natural environment of cats with successful predation to rodents. For this purpose, the prevalence of infection in cat-hunted and trap-captured *A. amphibius s.l.* was compared, revealing a significant higher prevalence in the cat-hunted animals (11.1% vs. 0%; Fisher's exact test: *p* = 0.0176), which would support the manipulation hypothesis. However, the challenge of such a field study is the choice of an adequate control group. In this study, the trap-captured rodents were not obtained from the same areas or during the same time frame as the cat-hunted group of rodents. Nevertheless, the fact that *T. taeniaeformis* infection was detected in both *Arvicola* groups (cat-hunted 22.6% vs. trap-captured 29.2%) suggests a widespread exposure to cat faeces. The lower prevalence of *T. gondii* infection in the trap-captured group, might be associated with a strong predation pressure. This could have led to an underestimation of the *T. gondii* infection rate in this group of rodents, as the *T. gondii* positive animals might rather have been easy prey for cats than captured in the traps (Berdoy et al., 2000; Vyas et al., 2007; Vyas and Sapolsky, 2010). Furthermore, most published studies performed on trap-captured rodents could also have underestimated the real prevalences (Table 5).

## 5 Conclusion

The present study is the first report of molecular detection and genetic characterization of *T. gondii* in cat-hunted small mammals in Switzerland. Our results revealed high levels of *T. gondii* infection in cat-hunted wild rodents, with Type II variant genotypes. In addition, a significant higher prevalence in cat-hunted vs. trap-captured rodents was found, providing additional evidence to support the behaviour manipulation hypothesis. However, the many factors at play in the natural environment, involving both predators and prey and the difficulty of obtaining large number of samples, highlights the need for additional studies that will have to include cat-hunted and trap-captured small mammals collected simultaneously at the same sites.

## Data availability statement

The original data presented in the study is included in the article (Suppl. Material 1), further inquiries can be directed to the corresponding author/s.

## Funding source declaration

This study was financially supported by the Institute of Parasitology of Bern, Switzerland.

## Ethics statement

For this study, we exclusively made use of animals collected for previously published studies, both for the cat-hunted small mammals (Geiger et al., 2022; Tschanz et al., 2011; Weinberger and Briner, 2021) and the trap-captured rodents (Burlet et al., 2011; Reperant et al., 2009; Stieger et al., 2002) (Suppl. Material 1).

## Declaration of competing interest

None.
