# Supplementary material for: High prevalence rates of Toxoplasma gondii in cat-hunted small mammals - Evidence for parasite induced behavioural manipulation in the natural environment?
Source: Int J Parasitol Parasites Wildl. 2023 Jan 23;20:108–16. doi: 10.1016/j.ijppaw.2023.01.007 (PMC9898578; doi:10.1016/j.ijppaw.2023.01.007)
Supplement: Multimedia component 2 [file mmc2.docx]

**SUPPLEMENTARY MATERIAL 2:** Prevalence of *Taenia taeniaeformis* in cat-hunted and trap-captured wild small mammals.

| **Small mammal group** | **Species** | **Prevalence (%) of *T. taeniaeformis*** | **No positive/no tested** |
| --- | --- | --- | --- |
| Group 1 (cat-hunted) | *Arvicola amphibius* | 28.6 | 2/7 |
|  | *Apodemus* sp. | 9.1 | 1/11 |
|  | *Myodes glareolus* | 0 | 0/6 |
|  | *Sorex* | 0 | 0/1 |
| Group 2: (cat-hunted) | *Arvicola amphibius* | 25 | 4/16 |
|  | *Apodemus* sp. | 0 | 0/14 |
|  | *Myodes glareolus* | 0 | 0/3 |
|  | *Crocidura russula* | 0 | 0/1 |
| Group 3: (cat-hunted) | *Arvicola amphibius* | 20 | 6/30 |
|  | *Apodemus* sp. | 0 | 0/17 |
|  | *Myodes glareolus* | 0 | 0/6 |
|  | *Microtus arvalis* | 12.5 | 1/8 |
| Group 4: (trap-captured) | *Arvicola amphibius* | 29.2 | 14/48 |
